# Do Surgical Smoke Evacuators Increase the Risk of Hearing Loss for Operative Personnel During Routine Adenotonsillectomy Surgery?

**DOI:** 10.7759/cureus.60214

**Published:** 2024-05-13

**Authors:** Taylor G Lackey, Jacqueline Rowley, Tiffany T Pham, Cory D Portnuff, Laylaa Ramos, Norman R Friedman, Brian W Herrmann

**Affiliations:** 1 Otolaryngology - Head and Neck Surgery, University of Colorado Anschutz Medical Campus, Aurora, USA; 2 Pediatric Otolaryngology, Children's Hospital Colorado, Aurora, USA; 3 UCHealth Hearing and Balance Clinic, UCHealth at University of Colorado Anschutz Medical Campus, Aurora, USA

**Keywords:** occupational medicine, surgical smoke evacuator, adenoidectomy, tonsillectomy, surgical smoke, sensorineural hearing loss, noise-induced hearing loss

## Abstract

Introduction: Aerosol mitigation equipment implemented due to COVID-19 has increased noise levels in the operating room (OR) during otolaryngological procedures. Intraoperative sound levels may potentially place personnel at risk for occupational hearing loss. This study hypothesized that cumulative intraoperative noise exposures with aerosol mitigation equipment exceed recommended occupational noise exposure levels.

Methods: Sound levels generated by the surgical smoke evacuator (SSE) during adenotonsillectomy were measured using a sound level meter and compared to surgery without SSE.

Results: Thirteen adenotonsillectomy surgeries were recorded. Mean sound levels with the SSE were greater than the control (72 ± 3 A-weighted decibels (dBA) vs. 68 ± 2 dBA; p=0.015). Maximum noise levels during surgery with SSE reached 82 ± 3 dBA.

Conclusion: Surgeons performing adenotonsillectomy with aerosol mitigation equipment are exposed to significant noise levels. Intraoperative sound levels exceeded international standards for work requiring concentration. Innovation is needed to reduce cumulative OR noise exposures.

## Introduction

Noise-induced hearing loss (NIHL) is the second-most common cause of sensorineural hearing loss [1]. Primarily affecting higher frequencies, NIHL can be caused by short-term exposure to high-intensity sound and/or long-term exposure to moderate sound levels [1-2]. Decibel hearing loss describes hearing loss in decibels that is unweighted. Occupational noise levels to predict hearing risk are often reported in the A-weighted decibels (dBA) scale as an eight-hour time-weighted average (TWA), as a better approximation of the human ear’s sensitivity to noise. High frequencies cause greater threshold shifts than low-frequency exposures of equivalent intensity, and the dBA scale relatively de-emphasizes low frequencies [3]. Examples of dBA sound levels are 35 dBA in the wilderness, 60 to 75 dBA for conversational speech, 78 dBA in a crowded urban apartment, 117 dBA in a loud dance, and 140 dBA for a jet aircraft at takeoff [4].

While United States governmental agencies have focused on delineating acceptable maximal sound exposure limits per day, to our knowledge, there is currently no maximum for ambient noise levels specific to intellectually demanding activities such as surgery. Although the National Institute for Occupational Safety and Health (NIOSH) of the Centers for Disease Control and Prevention recommends a noise exposure limit of 85 dBA, the Occupational Safety and Health Administration (OSHA) allows for noise exposure at 90 dBA over 8 hours [5-6]. The Environmental Protection Agency (EPA) recommends action when noise levels of 75 dBA are reached, not only to protect from NIHL but also to improve auditory comfort [7]. The permitted duration of sound exposure is halved for every 3 dBA increase above 85 dBA for NIOSH, while OSHA does so in 5 dBA increments above 90 dBA [8]. While these recommendations are generalized to all work environments, the International Standard Organization has provided guidance (ISO 11690-1:2020) that ambient noise levels should not exceed 35-45 dBA for activities that require concentration [8]. While this may not be achievable in the current operating room (OR), mitigation of noise can be useful, as surgeon performance decreases in tasked conditions with simulated noise of an average OR noise level with music (74 dB) compared to without music (65 dB) [9].

Studies examining occupational noise exposure in the OR prior to COVID-19 reported intraoperative sound levels averaging 65 dBA, with surgeons receiving greater exposure than anesthesiologists and other surgical staff due to their closer proximity to sound sources in the OR [9,10]. Procedures in the fields of urology, orthopedics, neurosurgery, and neurotology are at the highest risk for NIHL due to the noise-generating equipment used during these cases [10-14]. The pandemic reemphasized the importance of aerosol mitigation, with widespread implementation of surgical smoke evacuator (SSE) systems in many common otolaryngological procedures to reduce potential SARS-CoV-2 exposure [2,12,15]. A full understanding of the noise levels generated by these devices and their contribution to cumulative noise levels in the OR has been limited [3]. Seipp et al. evaluated five different SSE and demonstrated sound levels exceeded ISO guidelines for activities requiring concentrated thinking at higher power levels [16]. More recently, Grigoryan et al. [2] measured an SSE used in dermatologic practice at three different distances, demonstrating a sound level range of 73-88 dBA, which approximated or exceeded NIOSH and EPA guidelines [5,16]. Neither of these studies specifically addressed the cumulative noise effect when these devices are used in conjunction with other instrumentation during otolaryngological procedures.

To reduce the risk of COVID-19 exposure and other aerosolized respiratory viruses, our institution has utilized SSEs during aerosolizing procedures of the head and neck. We hypothesize that intraoperative noise levels generated by SSE devices exceed levels specified in their data sheets and that cumulative noise levels including SSE devices exceed recommended environmental noise limits during adenotonsillectomy.

## Materials and methods

**Figure 1:**
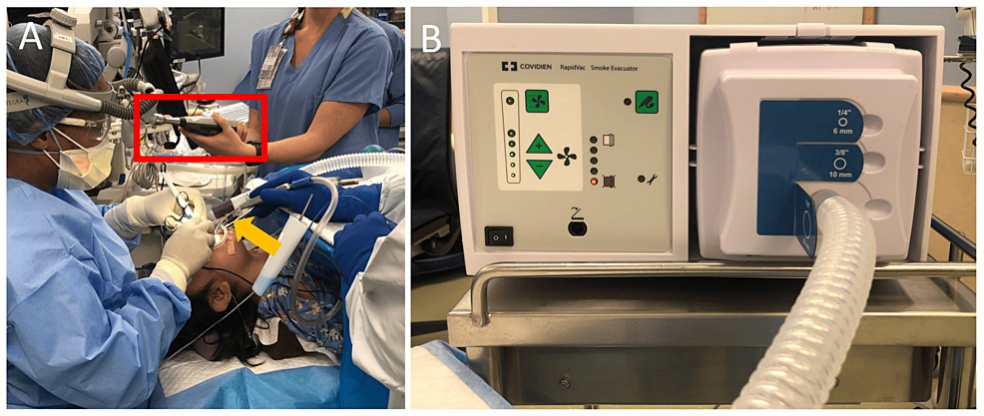
Setup of the SSE (A) OR setup with SSE. The yellow arrow points to the suction tip of the SSE, positioned next to the patient’s oral cavity. The red rectangle identifies the sound level meter positioned 16 inches equidistant from the surgeon’s shoulder. (B) SSE system with attached tubing. SSE: surgical smoke evacuator, OR: operating room

This was a prospective quality improvement observation study. Project approval was granted by the Organizational Research Risk and Quality Improvement Review Panel (approval number: 2009-4). This study was completed across 13 adenotonsillectomy (bilateral tonsillectomies with adenoidectomy) surgeries in three ORs with six different surgeons at a tertiary academic hospital from January 8, 2021, to August 1, 2023. All surgeries used the “portable” SSE on the maximum setting as per routine use prior to study implementation (COVIDIEN RapidVac Smoke Evacuator System, Medtronic, Minneapolis, USA; Figure 1). The SSE was comprised of a smoke evacuator and suction tubing.

Sound measurements were taken via the sound level meter (TSI Quest SoundPro SE/DL, WI, USA), which was comprehensively calibrated annually by e3 MSR West. Calibration of the microphone was performed before each test day. Recordings were performed 16 inches from the SSE, equidistant from the surgeon’s shoulder, for the duration of surgery [17]. The distance was determined to be the closest possible without compromising the sterile field or interrupting the team’s workflow. As controls, adenotonsillectomy surgeries were recorded without the use of the SSE. All adenotonsillectomies were performed with the use of a Bovie electrocautery, suction Bovie electrocautery, or coblater. Maximum suction with a Yankauer tip was used throughout the procedure. Music was not played during any surgeries so as to not confound sound levels.

The noise levels of the portable SSE and a “boom” style SSE (MAQUET LVAC, Rastatt, Germany) were measured on the maximum setting. The sound level of each device was measured in two empty ORs for two minutes and repeated for three trials. Noise levels were measured using the same sound level meter [17]. The mean and maximum noise levels during each measurement were recorded in equivalent continuous, A-weighted sound pressure levels (presented as dBA). Manufacturer websites of the devices were searched for datasheets, and sound level data was extracted if available.

Descriptive statistics were analyzed using SPSS Statistics version 28.0 (IBM Corp. Released 2021. IBM SPSS Statistics for Windows, Version 28.0. Armonk, NY: IBM Corp). The Shapiro-Wilk tests were used to test if the data set comes from a normal distribution. An unpaired two-tailed t-test was used to assess the differences between sound levels measured during surgery versus the control and to compare the portable and boom SSE sound levels. The statistical significance was set at p≤0.05.

## Results

**Figure 2:**
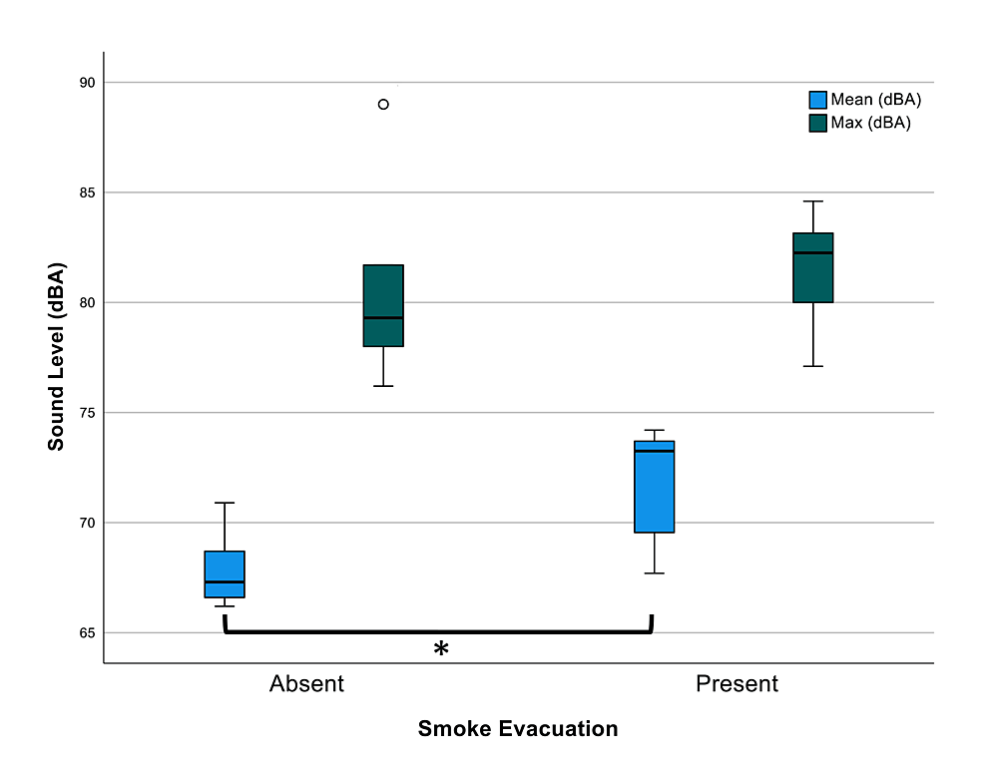
Sound levels (dBA) during adenotonsillectomy with and without SSE The mean sound levels generated during the SSE surgery were 72 dBA and 68 dBA without SSE (*p=0.015). The maximum sound levels during the SSE surgery reached 82 dBA compared to 81 dBA (p=0.732). Error bars represent calculated standard deviation. The circle represents an outlier for maximum sound levels without SSE and the upper standard deviation for this condition is flush with the box plot. SSE: surgical smoke evacuator, dBA: A-weighted decibels

A total of 13 adenotonsillectomy surgeries were recorded, eight with the portable SSE and five without the use of the portable SSE ("control"). The average duration of all surgeries was 16 ± 10 minutes standard deviation (SD). The mean sound level generated during the SSE surgery was 72 ± SD 3 dBA, which was significantly louder than the control of 68 ± 2 dBA (p=0.015). The maximum sound level during the SSE surgery reached 82 ± 3 dBA, which was not significantly louder than the control of 81 dBA ± 5 dBA (p=0.732; Figure 2).

**Figure 3:**
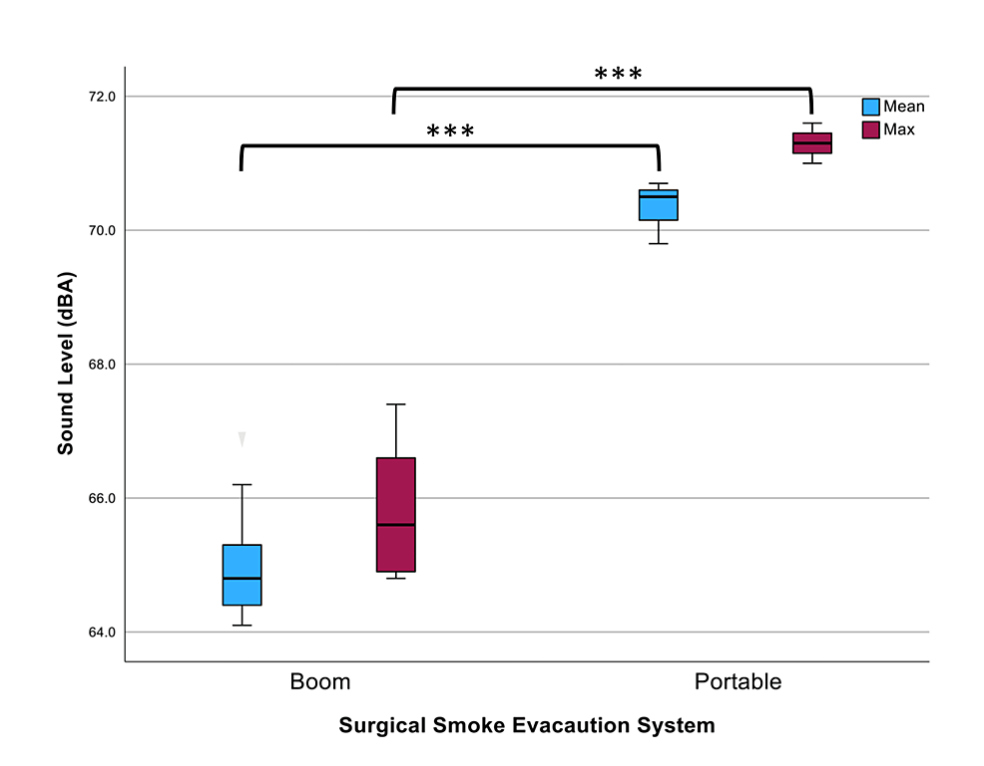
Sound levels (dBA) of the boom and portable SSE in a quiet OR Mean sound levels of 65 dBA and 70 dBA (***p<0.001) and maximum sound levels of 66 dBA and 71 dBA were found for the boom and portable SSE, respectively (***p<0.001). Error bars represent calculated standard deviation. SSE: surgical smoke evacuator, OR: operating room, dBA: A-weighted decibels

Testing of the portable and boom SSE in a silent OR demonstrated differing mean sound levels of 70 dBA and 65 dBA, respectively (p<0.001) and maximum sound levels of 71 dBA and 66 dBA, respectively (p<0.001) (Figure 3).

The investigation of maximum noise levels reported by manufacturer datasheets was 57 dBA for the portable and 68 dBA for the boom SSE, demonstrating the potential for incongruence between measured and manufacturer-reported sound levels, particularly with the portable SSE.

## Discussion

Adenotonsillectomy is considered a high-risk procedure for producing surgical aerosols with the potential for intraoperative transmission of the SARS-CoV-2 virus to OR personnel. This is attributed to the manipulation of tissue with a high viral load and the production of virus-containing vapors through the use of electrocautery [18]. In addition to the use of N95 masks and powered air-purifying respirators (PAPRs), mitigation of COVID-19 risk has included changes in operative equipment, including the introduction of SSEs. Already recommended by NIOSH to reduce the potential exposure of OR personnel to carcinogenic substances in surgical aerosols [15], these devices became standard equipment for common otolaryngological procedures as a mitigation strategy for COVID-19. In fact, during this study investigation, the state of Colorado instituted the use of SSEs for all procedures utilizing electrocautery. With the OR already known for potential excessive sound exposure, the inclusion of this equipment has added more sound into an already noisy environment, highlighting the need for an enhanced understanding of the potential risks for OR personnel hearing and communication [19-21]. This study data supported the hypothesis, suggesting that noise generated from SSE devices may exceed levels listed in their manufacturer datasheets. The determined total intraoperative sound levels were just below current United States agencies’ occupational noise limits, but they exceeded international standards for work requiring concentration.

To the authors’ knowledge, this is the first report evaluating noise levels in the OR during adenotonsillectomy using the required equipment for COVID-19 mitigation. While the maximum sound level generated during surgery with SSE (82 dBA) was not significantly different than the control (81 dBA), the mean sound level generated during surgery with SSE (72 dBA) was statistically significantly louder than the control (68 dBA). Peak levels are more likely contributed by variability in noise in the OR, possibly due to other non-SSE noise contributors such as other surgical equipment or communication. While the mean sound level was within acceptable limits for NIOSH and OSHA when extrapolating to a lower TWA for a day of surgery exposure, it did exceed ISO standards for work requiring concentration [7,8]. While reduction in OR noise may be difficult to achieve this ISO standard, 10 dBA can have notable effects on surgeon tasked performance as seen in Way et al. [9]. Furthermore, with noise exposure from surgical equipment close to the upper acceptable limit, any additional noise sources, such as music, may inadvertently increase the risk of intraoperative noise exposures for OR personnel above EPA and/or NIOSH recommendations [14].

Sound measurements of the portable SSE did not consistently align with noise levels reported from manufacturer specifications (71 vs. 57 dBA, respectively). A review of manufacturer data sheets identified that sound level measurements were performed at variable distances from the SSE, with some up to two meters, which may impact the noise levels recorded [2]. Two meters is an impractical distance for most otolaryngological procedures and underscores the need for diligence in understanding noise exposure risk in the OR. A difference of 14 dBA is significant in understanding the possible additive noise risk from SSE.

In addition to the concerns for NIHL from excessive intraoperative sound exposure, the deleterious effects of noise pollution (51-75 dBA) on intraoperative communication have been reported [10]. Background noise of 65-77 dB has been shown to reduce brain cognition and efficiency, and surgeons’ manual dexterity is reduced with noisy distraction [9,19,20]. Peaks of sound are associated with less case-relevant communication, and auditory processing decreases with an increase in noise [9,21]. This impaired communication is associated with an increased risk of adverse events [19,22]. The use of these personal protective equipment exacerbates the risk of impaired communication, as N95 and PAPR respirators decrease speech intelligibility [22]. Closed-loop conversation in the OR is recommended at all times, but especially in surgeries with greater noise pollution. It can be inferred from these various studies and our findings that sound pollution and intraoperative communication are impaired in aerosolizing surgeries with the use of safety equipment including SSE and respirators.

While current methods to reduce the sound levels in the OR may appear limited, there are other ways to help reduce the intraoperative risk of NIHL. Ensuring proper equipment function and minimizing the duration of high-noise surgical instrumentation can lessen cumulative sound exposure. Upgrading instruments with auditory advantages, such as suction devices that reduce noise emission without compromising function, is also a viable option to reduce noise levels in the OR [23]. This is an area of research and development in occupational medicine that may hold promise in reducing noise volume from suction, drills, and saws to create a quieter workplace. Reduction of extraneous background noise sources, such as music, has also been recommended [14].

The strengths of this study included conducting evaluations in a true OR environment and utilizing equipment used in patient care. Sound measurements were made in positions that accurately simulated the sound levels received by the otolaryngologist performing the procedure. The use of controls also enabled the most accurate method to assess the SSE noise levels and their contribution to overall noise levels during simulated procedures. Further investigation on methods to reduce noise in the OR and its impact on intraoperative communication is necessary.

The limitations of this study include its small size, variable confined to measuring live surgeries including different personnel, additional trainees, multiple operating room suites, and the limited number of SSE systems evaluated. While all standard noise sources normally present in the OR for adenotonsillectomy at our institution were included, this may not reflect background noise levels related to speaking, nor did it include any specialized surgical equipment based on surgeon preference. Results from this study may also not be generalizable to aerosol-generating procedures outside of otolaryngology.

## Conclusions

In our study, we found that adenotonsillectomies that utilized SSEs for aerosol mitigation had mean sound levels greater than those without SSEs. The mean sound levels with SSEs exceeded international recommendations for work requiring concentration. Real-world use of SSEs may expose the surgeon to higher volumes than manufacturer specifications and has the potential to negatively impact OR performance as well as NIHL. This study suggests further investigation into methods for sound mitigation is needed.
